# Kinetic Spectrophotometric Method for the 1,4-Diionic Organophosphorus Formation in the Presence of Meldrum′s Acid: Stopped-Flow Approach

**DOI:** 10.3390/molecules21111514

**Published:** 2016-11-11

**Authors:** Fatemeh Ghodsi, Sayyed Mostafa Habibi-Khorassani, Mehdi Shahraki

**Affiliations:** Department of Chemistry, University of Sistan and Baluchestan, P.O. Box 98135-674, Zahedan 9816744599, Iran; fa.ghodsi@yahoo.com (F.G.); smhabibikhorassani@yahoo.com (S.M.H.-K.)

**Keywords:** kinetics, zwitterionic intermediate, stopped-flow, mechanism

## Abstract

The kinetics of the reaction between triphenylphosphine (TPP) and dimethyl acetylenedicarboxylate (DMAD) in the presence of Meldrum’s acid (MA) for the generation of the 1,4-diionic organophosphorus compound has been investigated using the stopped-flow and UV-VIS spectrophotometry techniques. The first step of the reaction between TPP and DMAD for the generation of (I_1_) in ethanol was followed by the stopped-flow apparatus. This step was recognized as a fast step. The reaction between the intermediate (I_1_) and MA showed first-order kinetics, and it was followed by the UV-VIS spectrophotometry technique. The activation parameters for the slow step of the proposed mechanism were determined using two linearized forms of the Eyring equation. From the temperature, concentration and solvent studies, the activation energy (Ea = 20.16 kJ·mol^−1^) and the related activation parameters (ΔG^‡^ = 71.17 ± 0.015 kJ·mol^−1^, ΔS^‡^ = −185.49 ± 0.026 J·mol^−1^ and ΔH^‡^ =17.72 ± 0.007 kJ·mol^−1^) were calculated. The experimental data indicated that the reaction was zero-order in MA and second-order overall. The proposed mechanism was confirmed with the observed kinetic data obtained from the UV-VIS and stopped-flow techniques.

## 1. Introduction

**Figure 1:**
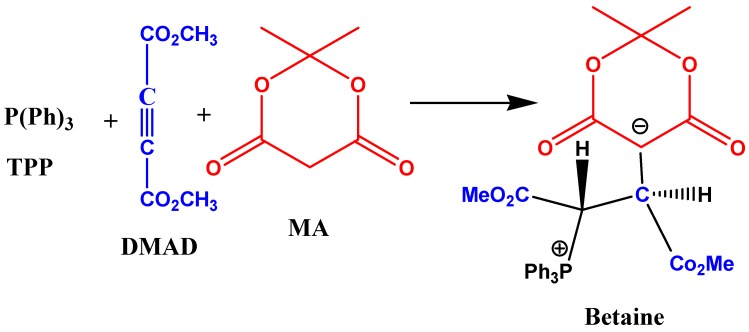
The reaction between triphenylphosphine (TPP) and dimethyl acetylenedicarboxylate (DMAD) in the presence of Meldrum’s acid (MA) for the generation of 1,4-diionic organophosphorus (betaine).

The trivalent phosphorus compound is known to be a nucleophile, whereas it behaves as an electron donor toward a good electron acceptor either in the ground or excited state [1,2]. In recent years, there has been increasing interest in the synthesis of organophosphorus compounds, i.e., those bearing a carbon atom bound directly to a phosphorus atom [3,4,5,6,7,8,9,10,11,12,13,14,15]. This interest has resulted from the recognition of the value of such compounds in a variety of biological, industrial and chemical synthetic uses [3,4,5]. A number of reactions have been observed that involve 1,4-diionic phosphorus compounds as elusive transient species [8,9,10]. In all of these reactions in which this diionic system was postulated, the betaines cannot be isolated, but appeared to occur as an intermediate on the pathway to an observed product. Researchers have recently described the synthesis of stable diionic compounds from the reaction between triphenylphosphine (TPP) and electron-deficient acetylenic esters in the presence of NH, OH, SH and CH acids [11,12,13,14,15,16,17,18,19,20,21,22,23,24,25,26,27,28]. The synthesis reaction of triphenylphosphine, acetylenedicarboxylic acid (ADA) and carbazole for the preparation of the crystalline and stable 1,5-diionic organophosphorus compound has been reported earlier [12]. In addition, the kinetics of these reactions has been published recently [29,30]. Numerous kinetic studies have been reported by the stopped-flow method [31,32]. In our previous works, the applications of the stopped-flow technique in kinetic and mechanistic studies have been reported for some organic synthesis reactions as cyclic crystalline phosphorous and pyrrole phosphorous ylides [33,34]. Herein, we describe the kinetic results along with the detailed mechanistic studies of the reaction between triphenylphosphine (TPP) and dimethyl acetylenedicarboxylate (DMAD) in the presence of Meldrum’s acid (MA) for the generation of the 1,4-diionic organophosphorus compound (betaine; Figure 1). The synthesis of this compound has been reported previously [12].

Meldrum’s acid (MA) and 1,3-dimethylbarbituric acid (BA) have considerably higher acidity (pK_a_ = 7.3–7.7 in DMSO) than acyclic analogues, such as dimethylmalonate (pK_a_ = 15.9 in DMSO), or even the diketone analogues, such as 5,5 dimethylcyclohexane-1,3-dione (pK_a_ = 11.2 in DMSO) [35]. Thus, the second acidic C–H of MA or BA can undergo a proton-transfer reaction to convert the stable ylide (k_4_, Step 4) to the 1,4-diionic organophosphorus compound (betaine; Figure 1). The individual property of MA in comparison with other analogues causes significant changes and newness in the reaction mechanism. The origin of the anomalously high acidity of Meldrum’s acid has been the subject of recent theoretical studies [36,37,38]. In this work, experimental studies are undertaken by the stopped-flow and UV-VIS spectrophotometry techniques.

## 2. Results and Discussion

### 2.1. Spectral and Kinetic Studies of the Overall Three-Component Reaction by the UV-VIS Spectrophotometry Technique

To gain a better understanding of the reaction mechanism between TPP, DMAD and MA, a kinetic study of the reaction was carried out using the UV-VIS spectrophotometry technique. The UV-VIS spectrum was measured over the concentration range 1.0 × 10^−3^ M ≤ M product ≤1.0 × 10^−2^ M to confirm a linear relationship between the absorbance and concentration values.

**Figure 2:**
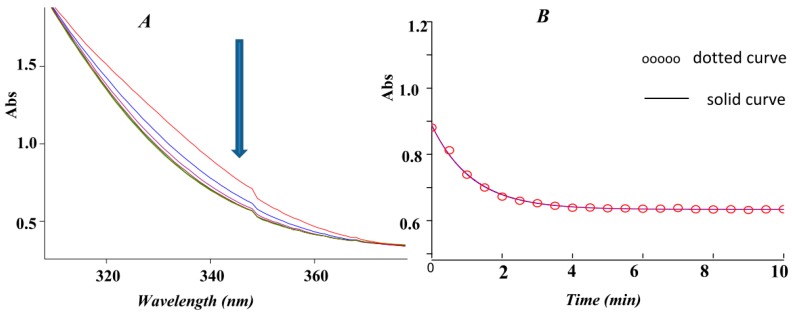
(**A**) The UV-VIS spectra for the reaction between TPP (5.00 × 10^−3^ M), DMAD (5.00 × 10^−3^ M) and MA (5.00 × 10^−3^ M); the downward direction of the arrow indicates the reaction progress; (**B**) The original experimental absorbance change (dotted curve) along with the second-order fit curve (solid curve) against time at 320 nm and 20.0 °C in ethanol.

It was necessary to find the appropriate wavelength in order to follow the kinetics of the reaction. For this purpose, a 0.3-mL aliquot of a 1.5 × 10^−2^ M solution of reactants TPP and MA in ethanol solvent was pipetted into a quartz spectrophotometer cell, then a 0.3-mL aliquot of a 1.5 × 10^−2^ M solution of reactant DMAD was added to the mixture according to the stoichiometry of each reactant in the overall reaction. The reaction was monitored by recording scans of the entire spectra with 1-min intervals during the whole reaction time at 20 °C (Figure 2A). The downward direction of the arrow indicates the reaction progress. The recorded relevant spectrum of each compound, TPP, DMAD and MA, in ethanol had no disturbance with the UV-VIS spectra of the reaction progress (360–380 nm).

**Scheme 1:**
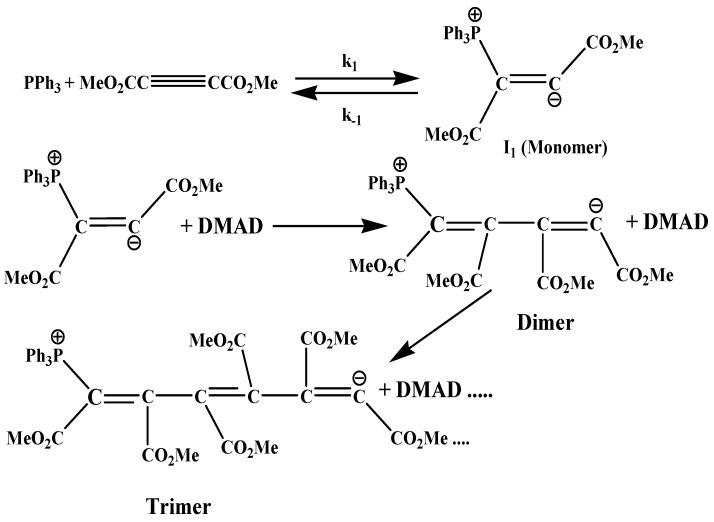
The reaction between TPP and DMAD.

**Figure 3:**
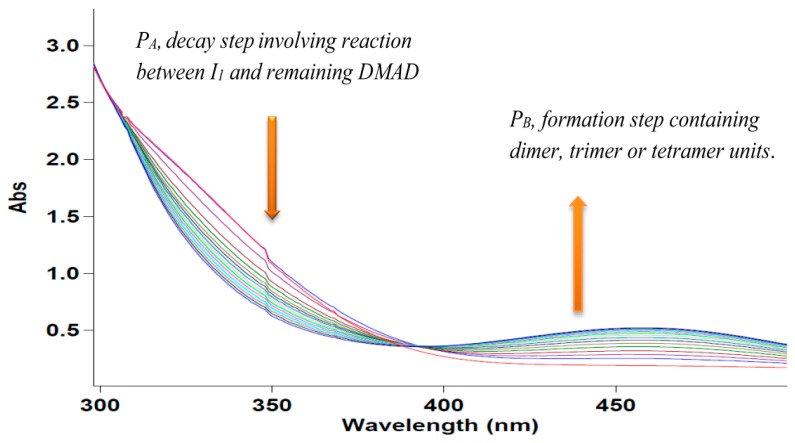
UV spectra of a binary mixture with TPP (2.50 × 0^−3^ M) and DMAD (5.00 × 10^−3^ M). The downward direction of the arrow indicates Part A or the decay step involving the reaction between I_1_ and the remaining DMAD. The upward direction of the arrow indicates the formation of the product in Part B that is tentatively presumed to contain polymeric oligomers (e.g., dimer, trimer; Scheme 1).

A series of two-component reactions between TPP and DMAD, TPP and MA and also DMAD and MA was monitored separately to find which reagents were present at the beginning of the reaction (Step 1). At first, a 0.500-mL aliquot of a 5.00 × 10^−3^ M solution of reactants TPP and a 0.500-mL aliquot of a 1.00 × 10^−2^ M solution of DMAD (to generate intermediate **1** (I_1_) in ethanol) were pipetted into a quartz spectrophotometer cell, and the mixture was monitored by recording scans of the entire spectra. It was found that the reaction between TPP and DMAD leads to I_1_ (Scheme 1) in methanol, and the other binary mixture did not show any changes in the studied wavelength and time ranges. Herein, intermediate **1** (I_1_) is generated rapidly (could not be seen by the UV-VIS instrument), which subsequently reacts with the remaining DMAD (decay step; Figure 3, Part A) to form a few large conjugated molecules (formation step; Figure 3, Part B) in accord with the following reaction (Scheme 1) [34]. As a result, in the reaction mechanism between TPP, DMAD and MA, Step 1 starts with reactants TPP and DMAD for generating I_1_.

The spectra of binary mixture (TPP and DMAD) showed that the intermediate was produced in Step 1 of the wavelength range of 300–320 nm (Part A, Figure 3). Hence, appropriate wavelengths were discovered to be 320, 330 and 350 nm, corresponding mainly to the I_1_. The chosen wavelength of 320 nm gave us the chance to find the practical conditions that allow a kinetics and a mechanistic investigation of the reaction. Herein, in all of the experiments, the UV-VIS spectrum of the product was measured over the concentration range 1.00 × 10^−3^ M ≤ product M ≤ 1.00 × 10^−2^ M to confirm a linear relationship between the absorbance and concentration values. Further experiments for the detection of intermediate I_1_ follow in the section below.

### 2.2. Stopped Flow Apparatus

#### Order of the Fast Step

Since the production of I_1_ was fast, we investigated this Step 1 using the stopped-flow apparatus. In a typical single-mixing experiment, one syringe contained 5.00 × 10^−3^ M of TPP mixed with 5.00 × 10^−3^ M of MA (since there is no reaction between them), and the other syringe contained 5.00 × 10^−3^ M of DMAD in the ethanol solvent. For each run, equal volumes of both solutions were mixed in the mixing chamber, and the changes in the absorbance were monitored at 320 nm during a chosen period of time. Rate constants are presented as an average of several kinetic runs (at least 6–10) and were reproducible within a ±3% margin (see Table 1).

In the stopped-flow instrument, the temperature of the reaction was maintained to within ±0.1 °C at various temperatures using a circulating water bath.

**Figure 4:**
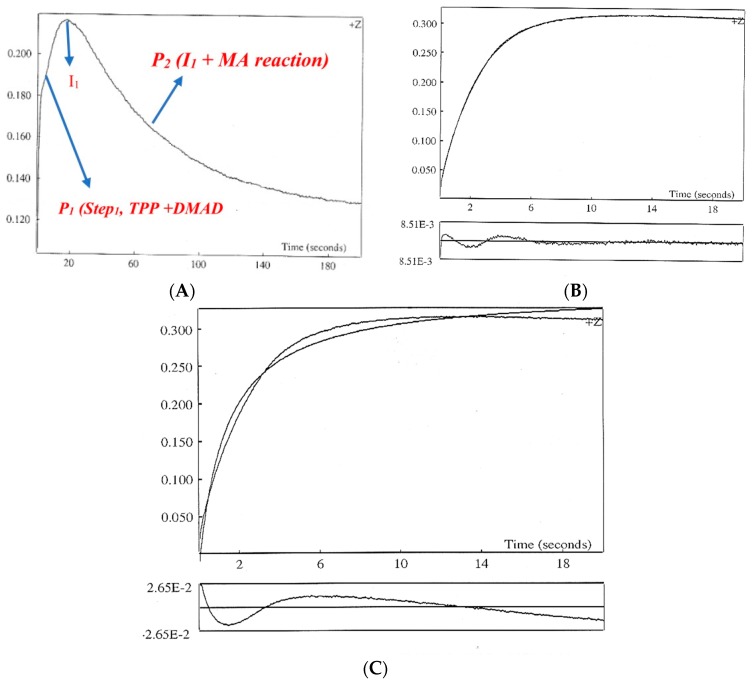
Analyzed kinetic traces for the reaction between TPP, DMAD and MA: (**A**) the stopped-flow spectra at 320 nm and 20 °C in ethanol involving Parts P_1_ (TPP + DMAD reaction) and P_2_ (I_1_ + MA reaction); (**B**) second-order fit curve for the expanded part (P_1_, Step 1, k_1_), which exactly fits to the original experimental curve at 320 nm and 20 °C in ethanol using the stopped-flow technique; (**C**) first-order fit curve for the expanded part (P_1_, Step 1, k_1_), which does not fit appropriately to the original experimental curve at 320 nm and 20 °C. P_1_ is relevant to the reaction between TPP and DMAD for the generation of I_1_. P_2_ is relevant to the reaction between I_1_ and MA.

**Figure 5:**
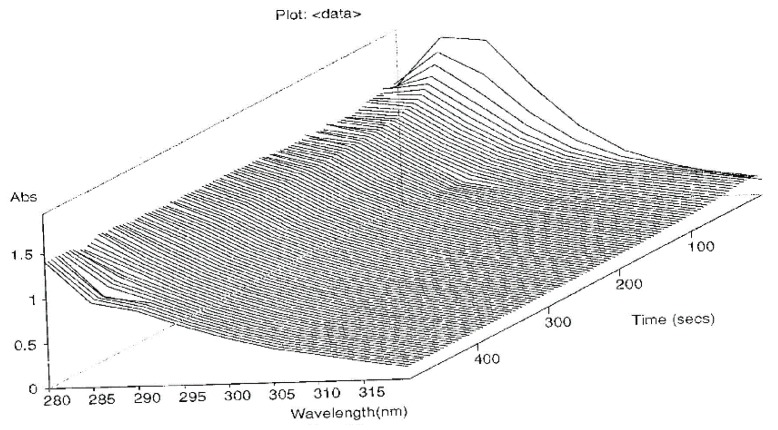
Time-resolved spectra (many spectra are obtained in the 280–320-nm range per shot with the 0.15-s interval) of 5.00 × 10^−3^ M of each compound mixed together in ethanol at 20 °C.

Figure 4A,B displays a typical kinetic trace at 320 nm generated from a number of spectra (see Figure 5). As can be seen, upon mixing of the reagents, an increase in the absorbance peak appears at 320 nm, which then decreases slowly in intensity. Each reactant alone (TPP, DMAD or MA) has no absorbance peak at 320 nm. Therefore, the time-resolved spectrum in Figure 4A,B provides clear evidence for the formation and decay of the intermediate (I_1_), the spectrum of which was characterized by an absorbance at 320 nm. At first glance, according to the graph recorded using the stopped-flow apparatus, the speed of reaction between TPP and DMAD for the creation of intermediate I_1_ is much faster (k_1_; Part P_1_, Figure 4A,B) than the speed of the reaction involving the consumption of intermediate **1** (I_1_) and MA (Part P_2_, Figure 4A). The intermediate is produced in much less time (20 s) and decays in a longer time (over 180 s). It can be concluded that the rate-determining step for the overall reaction depends on the consumption of the intermediate in Part P_2_ (longer time; Part P_2_, Figure 4A). Figure 4B refers to the second-order fit curve for expanded Part P_1_ (Step 1, k_1_, reaction between TPP and DMAD), which exactly fits to the original experimental curve at 320 nm and 20 °C. Under the pseudo-order conditions, the rate of the reaction was found to be first-order with respect to TPP or DMAD. In this case, the concentration of TPP was 1.00 × 10^−2^ M and the concentration of DMAD was selected as 5.00 × 10^−3^ M.

Step 1, which is associated with an increase in absorbance, was analyzed on a short time scale at 320 nm. It may be noted here that the single exponential analysis of this step (growth) resulted in almost identical rate constant values; however, it is a safer practice to analyze such kinetic traces (Figure 4A,B) using a double exponential function in order to obtain the rate constant for the first reaction step more accurately.

The rate constant of the fast step (k_1_) of the reaction between TPP and DMAD in ethanol solvent (24.7 D) has been reported in Table 1.

### 2.3. UV-VIS Experiments

#### Overall Order

The reaction between compounds TPP and DMAD leads to I_1_, which the stopped-flow spectrophotometry recognizes with a completion time of about 20 s. This time is too short and could not be detected using the UV-VIS technique (see Figure 4B), but the subsequent step (decay, longer time, P_2_), which is slower than the first step and is relevant to the reaction between I_1_ and MA, was investigated using the UV-VIS method (Figure 4A, P_2_).

With a 5.00 × 10^−3^ M concentration of each reactant (TPP, DMAD and MA with 5.00 × 10^−3^ M), the experimental absorbance curve was recorded versus time at 20 °C and wavelengths of 320 nm. This graph can be seen in Figure 2B. The original experimental absorbance curve (dotted curve) of the consumption of intermediate **1** using UV-VIS spectrophotometry exactly fit the first-order curve (solid curve). It was obvious that the overall order of consumption of intermediate **1** and reactant MA was one. Therefore, the rate law can be written as:

Rate = k_obs_ [I_1_]^z^ [MA]^γ^ (1)

Therefore: z + γ = 1(2)

### 2.4. Effect of Concentration

With the purpose of finding the partial order of I_1_ and reactant MA under pseudo-order conditions, another experiment was designed. In this experiment, 5.00 × 10^−3^ M of reactant TPP, 5.00 × 10^−3^ M of reactant DMAD and 5.00 × 10^−2^ M of reactant MA were used. The rate law for the new experiment can be written as:

Rate = k_obs_′ [I_1_]**^z^** (3)

k_obs_ = k_ove_′ [MA]^γ^ (4)

In this experiment, the original experimental absorbance curves versus times provided a pseudo-first order. The experimental absorbance curve versus times along with a first-order fit for this experiment was recorded at 320 nm and 20.0 °C. Then, the rate constant of the reaction was automatically obtained using the software program [39]. Herein, according to Equation (3), the partial order with respect to intermediate **1** was 1 (z = 1).

As a result of the two experiments by UV-VIS experiments, z = 1 on the basis of new experiment and z + γ = 1 according to the first experimental; therefore, the partial order of compound MA can be determined as zero (γ = 0), and the experimental rate law can be expressed as:

Rate = k_ovr_ [I_1_] [MA] (5)

Rate = k_obs_ [I_1_] [MA] (6)

k_obs_ = k_ovr_ (7)

From the stopped-flow experiments, the order of reaction with respect to TPP (α) and DMAD (β) was deduced as two (α + β = 2). Thus, the overall order of the reaction is two with respect to the results obtained using the stopped-flow and UV-VIS techniques.

### 2.5. Effect of Solvent and Temperature

In order to determine the effect of temperature and solvent environment on the overall reaction rate, previous experiments were repeated under different temperatures and solvents. For this purpose, 1,2-dicholoroethan (10.37 D) was used as another solvent in the experiment. The results showed that the rate of the overall reaction speeds up in the 1,2-dicholoroethan solvent with a low dielectric constant compared to the higher dielectric constant of ethanol (24.7 D). The obtained rate constants for the overall reaction are listed in Table 2 under different temperatures and solvents.

**Figure 6:**
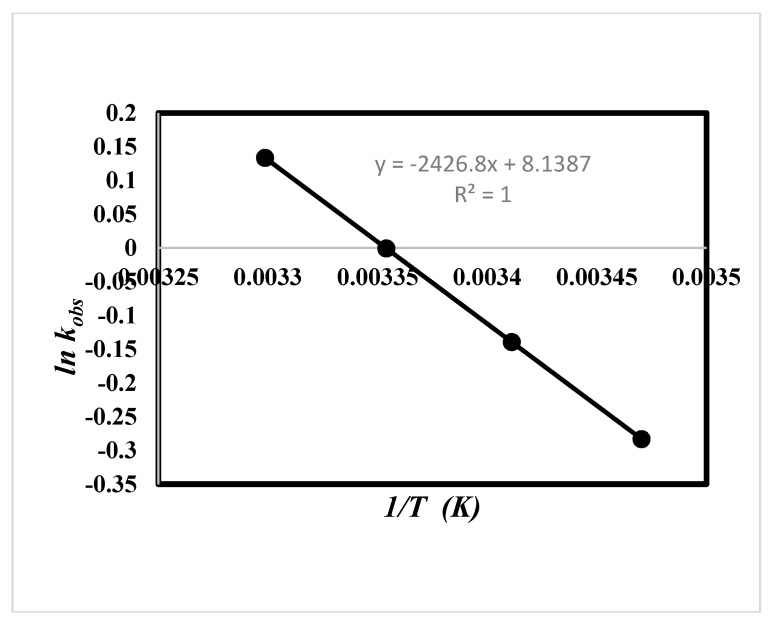
The dependence of the observed rate constant (ln k_obs_) on the reciprocal temperature for the reaction between compounds TPP, DMAD and MA in ethanol at a wavelength of 320 nm.

As can be seen in Table 2, the rate of reaction increases in two solvents at higher temperatures. Furthermore, the decreasing rate constants when using high dielectric solvents indicated that the activated complex must have less charges than the reactant at the rate-determining step; therefore, the solvent stabilized the reactant more than the activated complex. In other words, the reactants were solvated to a greater extent in comparison to the activated complex, and this reduces the rate and increases the activation energy in high dielectric solvent (see Table 2; T = 293.15). In the studied temperature range, the first-order rate constant (ln k_obs_) of consumption of I_1_ was inversely proportional to the temperature, which is in agreement with the Arrhenius equation. This behavior is shown in Figure 6. The activation energy, for the reaction between TPP, DMAD and MA in ethanol was calculated (Ea = 20.18 kJ/ mol) from the slope of Figure 6.

**Figure 7:**
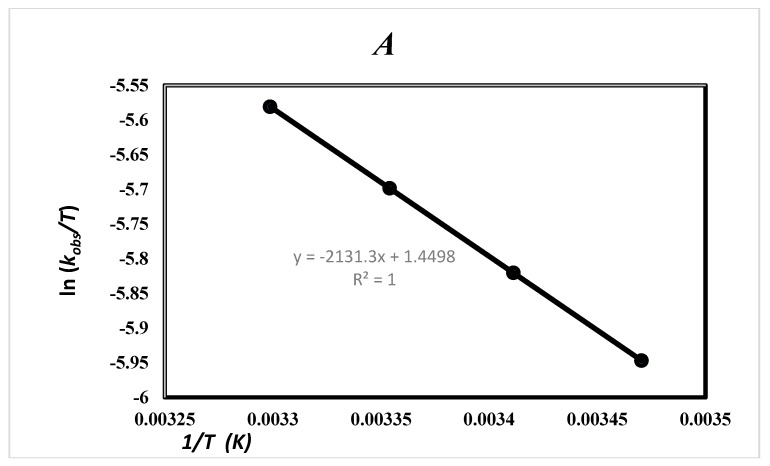

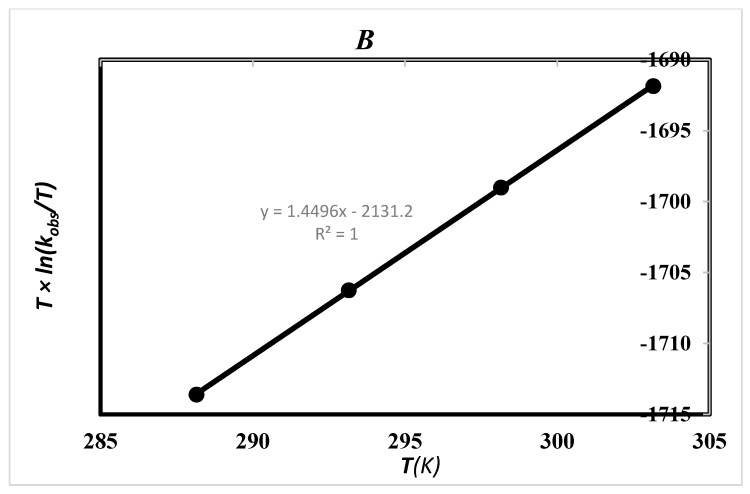
Eyring plots, for the reaction between TPP, DMAD and MA in ethanol at a wavelength of 320 nm: (**A**) according to Equation (8); (**B**) according to Equation (9).

The activation parameters, which involve ΔG^‡^, ΔS^‡^ and ΔH^‡^, can now be calculated on the basis of the Eyring equation (Figure 7A; Equation (8)). Figure 7B shows a different linearized form of the Eyring equation (Equation (9)). The standard errors for the activation parameters have been calculated and reported along with these parameters for both used forms of the Eyring equation in Figure 7A,B [40].

(8) $$\ln\left(\frac{k}{T}\right)=-\frac{\Delta H^{‡}}{\mathrm{RT}}+\left(\frac{\Delta S^{‡}}{R}+\ln\frac{K_{b}}{h}\right)$$

(9) $$\mathrm{Tln}\;(\frac{k}{T})=\left(-\frac{\Delta H^{‡}}{R}\right)+T\left(\frac{\Delta S^{‡}}{R}+\ln\frac{K_{b}}{h}\right)$$

The values of calculated activation parameters (ΔS^‡^, ΔH^‡^ and ΔG^‡^) have been listed for the reactions between TPP, DMAD and MA in ethanol at 293.15 K, using Equations (8) and (9), respectively, in Table 3.

The activation enthalpy (ΔH^‡^) and the activation Gibbs free energy (ΔG^‡^) were positive, which suggest that the energy required for the reaction was relatively high, so the reaction was chemically controlled. However, the negative value of the activation entropy (ΔS^‡^) shows that energy must be partitioned into a lesser state at the TS. Therefore, the transition state in the region of the activated complex had a more ordered or more rigid structure, which indicates an associative mechanism. The reaction is entropy controlled because the activation enthalpy (ΔH^‡^) was less than TΔS^‡^.

**Scheme 2:**
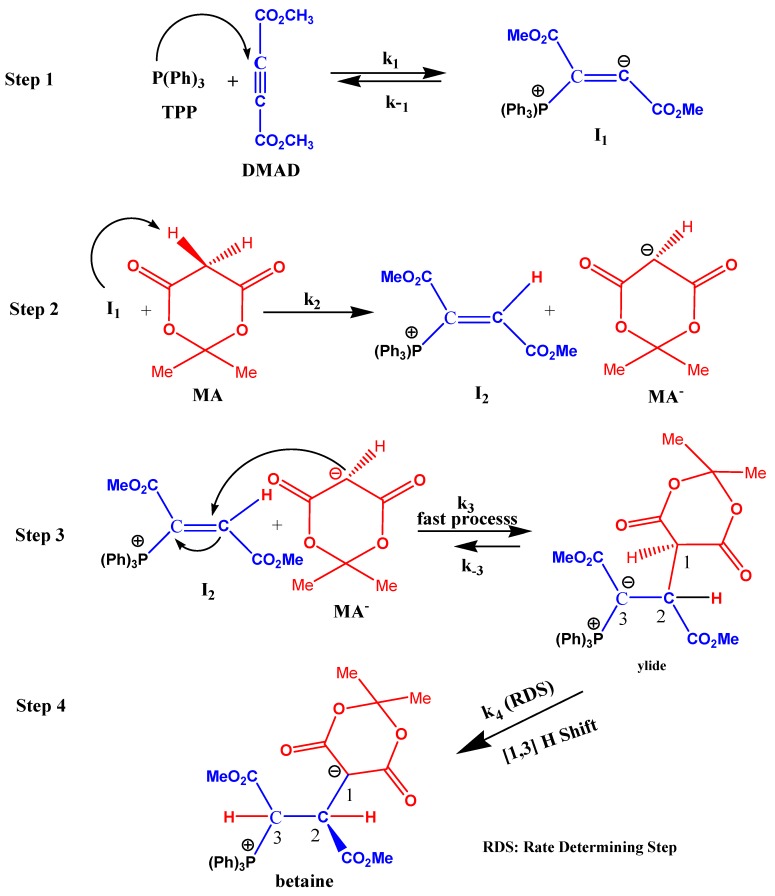
The proposed mechanism of the three-component reaction in the ethanol solvent.

The data obtained from the experiments with the UV spectrophotometry method were not able to determine the rate determining step. From solvent studies, both Step 2 (rate constant, k_2_) and Step 4 (rate constant, k_4_) had a good potential to be the rate-determining step (Scheme 2). Because, reactants in Step 2 or Step 4 (phosphonium ion (I_1_) or ylide, respectively) carried full charges and can form more powerful ion-dipole bonds to the solvent with high dielectric constant while their transition-state (TS_2_ or TS_4_) had partial charges and the effect of the solvent for stabilizing them was less stronger than the reactants, so the rate of the reaction sped up in the presence of solvent with high dielectric constant. These observations for Step 2 and Step 4 as the possible rate-determining steps were reverse for the solvent with a low dielectric constant (see Table 3).

### 2.6. Global Kinetic Analysis of Data for the Two- and Three-Component Reactions Using the Pro-K Software

Once a full set of data was acquired and saved to disc by the stopped-flow instrument, Pro-K global analysis software can then be used to test different reaction schemes and provide a more accurate gauge of the rate constants by analyzing all kinetic records simultaneously [41,42]. Pro-K involves an equation editor in combination with numerical integration techniques to fit reaction models to data. Moreover, the Pro-K analyzer yields singular value decomposition (SVD), which is a mathematical process that produces a set of basis spectra, time-dependent amplitudes and singular values. SVD suggests the minimum number of spectrally-distinct reaction components, which are present in the reaction and help in proposing a reaction mechanism [41,43].

A 3D display of the absorbance changes versus time at a 280–320-nm range (Figure 5) obtained by Pro-K software resulting from rapid mixing of TPP and DMAD was recorded. Many possible models with the initial concentrations of components were entered in the entry gate. The values of the rate constants analyzed by using the SX-18MV v4.47 operation confirmed that the equilibrium reaction model (TPP + DMAD <> I_1_) for Step 1 is the most reasonable mechanism. Furthermore, the 3D residual plot following the fitting of the time courses of spectrum confirmed the equilibrium reaction model (TPP + DMAD <> I_1_).

**Figure 8:**
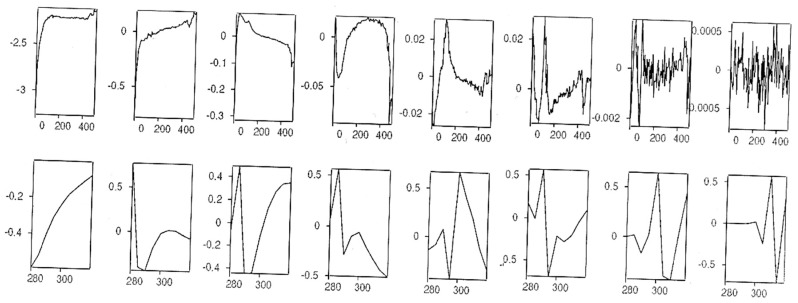
Basis spectra and time-dependent amplitudes derived from singular value decomposition (SVD) of the experimental matrix (only the first eight vectors displayed.)

Moreover, considering the set of basis spectra (Figure 8), time-dependent amplitudes and singular values decompositions (22.613, 1.284, 0.518, 0.238, 0.106, 0.080, 7.91 × 10^−3^ and 2.46 × 10^−3^) of the two-component reaction derived by the Pro-K analyzer, the presence of three species assigned as TPP, DMAD and I_1_ was confirmed; Columns 4–8 showing only noise (Figure 8).

Furthermore, a reaction model was evaluated for the three-component reaction in the simple form of steps as TPP + DMAD <> I_1_, I_1_ + MA > I_2_, I_2_ + MA^−^ > ylide and ylide > betaine. Due to the complexity of the three-component reaction, the fitted curves were not reasonable; however, by inspection of the SVD analysis, it can be concluded that there are only six significantly-changing species assigned (like Figure 8 for the two-component reaction between TPP and DMAD) as TPP, DMAD, Meldrum’s acid (MA), I_1_ and two other intermediates (**I_2_** and MA^−^) in accordance with the following proposed mechanism.

### 2.7. Mechanism Discussion

The plausible mechanism is exhibited schematically in Scheme 2 in accordance with the reports in the literature [44,45,46,47] and the experimental data by the UV-VIS and stopped-flow techniques. At the first step (k_1_), the nucleophilic addition of triphenylphosphine TPP to DMAD as a Michael acceptor occurs, which generates the carbene-ylide intermediate (I_1_) [48,49]. Then, in Step 2, I_1_ is protonated by Meldrum’s acid (MA). At the next step (k_3_), the positively-charged ion (**I_2_**) is attacked by the conjugate base (MA^−^) of Meldrum’s acid to create phosphorane (ylide). Finally, with an intra-molecular proton-transfer reaction in Step 4 (k_4_), the desired product was obtained. The solvent effect indicates that the reaction rate decreases in solvents with high dielectric constants; this becomes possible when the dipolar compound (ylide, an ionic compound in the ground step) stabilizes much more than the activated complex with partial charges in transition state (TS_4_), as well as TS_2_ in Step 2. Therefore, Step 2 and Step 4 have a good potential for being the rate-determining steps on the basis of the obtained results from the solvent study on the reaction environment. This decision needs more documents that will be provided by an inside view of the reaction mechanism in the section below.

If Step 2 (k_2_) is the rate-determining step, the general rate law can be written as follows:

(10) $$\mathrm{Rate}=\frac{k_{1}k_{2}\left[\mathrm{TPP}\right]\left[\mathrm{DMAD}\right]\left[\mathrm{MA}\right]}{k_{-1}+k_{2}\left[\mathrm{MA}\right]}$$

The following assumption is logical under the above condition:

k_−1_ >> k_2_ [MA] (11)

Therefore, the rate law becomes:

(12) $$\mathrm{Rate}=\frac{k_{1}k_{2}}{k_{-1}}\left[\mathrm{TPP}\right]\left[\mathrm{DMAD}\right]\left[\mathrm{MA}\right]$$

This equation, which is of third-order kinetics, is not in agreement with the experimental results (second-order kinetics). For this reason, Step 2 could not be a rate-determining step.

When Step 4 is a rate-determining step, the general rate law can be expressed:

(13) $$\mathrm{Rate}=\frac{k_{1}k_{4}\left[\mathrm{TPP}\right]\left[\mathrm{DMAD}\right]}{k_{-3}+k_{4}}$$

Under the above condition, k_4_ << k_-3_, then the rate law can be stated as:

(14) $$\mathrm{Rate}=\frac{k_{1}k_{4}\left[\mathrm{TPP}\right]\left[\mathrm{DMAD}\right]}{k_{-3}}\;\mathrm{or}$$

(15) $$\mathrm{Rate}=k_{\mathrm{obs}}\left[\mathrm{TPP}\right]\left[\mathrm{DMAD}\right],\;k_{\mathrm{obs}}=\frac{k_{1}k_{4}}{k_{-3}}$$

The recent equation is of second-order kinetics and compatible with the experimental data from the UV-VIS and stopped-flow techniques, as well as the solvent studies on the reaction environment. In addition, more possibility is attributed to Step 4 for being the rate-determining step; herein, the proton transfer is undertaken by the second acidic CH of Meldrum’s acid (MA) with an intra-molecular process. This transferring (Step 4) is more difficult and slower than the proton transfer of the first acidic C–H of MA (Step 2) with an inter-molecular process. Step 4 was found to be the rate-determining step. In previous work with TPP, DMAD and 3,5-dimethylpyrazole in ethoxyethane (diethyl ether), the first step (the formation of I_1_) was a rate-determining step [24]. In the current work, Step 4 (1,3-protropic rearrangement) was a rate-determining step due to it being able to undergo the proton transfer reaction to convert the stable ylide (k_4_, Step 4) to the 1,4-diionic organophosphorus compound (betaines; Scheme 2). The ylide prepared by the reaction between TPP, DMAD and MA was isolated and characterized previously [50]. On the other hand, the ylide formed with malononitrile (pK_a_ = 11.2 in water) does not undergo a 1,3-prototrophic rearrangement [51], whereas with MA, it does. It seems to be a matter of difference in the acidity of the attached carbanionic moieties. Therefore, some solvents, such as dinitromethane (pK_a_ = 3.6 in water) [52], may undergo the 1,3-proton shift, give the betaines and yield a similar overall rate constant in ethanol. Step 4 is likely an over-simplification, and it implies a concerted process; such processes are rare [53] and, further, when deuterated solvent is used, show varying degrees of intramolecularity [50].

## 3. Chemicals and Apparatus

The TPP, DMAD, MA and solvents were obtained from Merck (Darmstadt, Germany) and Fluka (Buchs, Switzerland) and used without further purifications. The stopped-flow apparatus (Model SX-18 MV, Applied Photophysics Company, Surrey, UK) and the associated computer system are from Applied Photophysics Company, (Surrey, UK). For a total of 100 μL/shot into a flow cell with 10-mm light path, the fastest time for mixing solutions and recording the first data point is about 5 ms. Kinetic traces are taken at different wavelengths between 320 and 250 nm, and the data are analyzed with the SX-18MV v4.47 operation (Applied Photophysics, Surrey, UK), which allows the synthesis of artificial sets of time-dependent spectra, as well as spectral analysis of the intermediates. In a typical single-mixing experiment, one syringe contains 5.00 × 10^−3^ M (18.6 × 10^−3^ mL) DMAD or mixed with 5.00 × 10^−3^ M (0.022 g) MA for the three-component reaction, and the other contains 5.00 × 10^−3^ M (0.0200 g) TPP in ethanol solvent. For each run, equal volumes of solutions are mixed in the mixing chamber, and the changes of the absorbance are monitored during a chosen time. Rate constants are presented as an average of several kinetic runs (at least 6–10) and are reproducible within ±3%. Furthermore, the overall reaction is followed by monitoring absorbance changes of the product (betaine) with time on a Varian (Model Cary Bio-300, Palo Alto, CA, USA) UV-VIS spectrophotometer (Palo Alto, CA, USA) with a 10-mm light-path cell. In the two types of instruments, the temperature of the reaction is maintained to within ±0.1 °C at various temperatures by a circulating water bath.

## 4. Conclusions

(a) The reaction mechanism starts with a fast reaction between reactants TPP and DMAD. This step was recognized using the stopped-flow technique. The obtained result showed that the order of reaction with respect to each reactant (TPP or DMAD) is one.

(b) The consumption of I_1_ and MA was studied using UV-VIS and followed first-order kinetics. The partial order of compound MA was determined to be zero, and its concentration had no effect on the rate of the reaction.

(c) The rate of overall reaction decreases in solvents with higher dielectric constants, so at the rate-determining step, the activated complex must have less charges than the reactant. Therefore, the solvent stabilizes the reactant more than the activated complex (Step 4, Scheme 2). The effect of solvents on the overall reaction was contrary to the first step (fast step).

(d) The kinetic data and solvent study on the reaction environment experimentally showed that Step 4 of the proposed mechanism is a rate-determining step.

(e) Investigations of the consumption of I_1_ at different temperatures allowed the determination of the activation parameters with respect to the slow step of the proposed mechanism. From the temperature, concentration and solvent studies, the activation energy (Ea = 20.16 kJ·mol^−1^) and the related activation parameters (ΔG^‡^ = 71.17 ± 0.015 kJ·mol^−1^, ΔS^‡^ = −185.49 ± 0.026 J·mol^−1^ and ΔH^‡^ = 17.72 ± 0.007 kJ·mol^−1^) were calculated.

(f) The reaction is entropy controlled because the TΔS^‡^ is much greater than activation entropy (ΔH^‡^).

(g) The high positive values of activation Gibbs free energy (ΔG^‡^) suggest that the reaction is chemically controlled.

(h) High negative values of the activation entropy express that the activated complex has a more ordered or more rigid structure, which indicates an associative mechanism.

(i) The obtained general rate law from the proposed mechanism indicates that the overall reaction is second-order, and it depends on the concentration of the compounds TPP and DMAD. This is compatible with the observed kinetics data obtained from both the stopped-flow and UV-VIS studies.

## Figures and Tables

**Table 1:** Kinetic data for Step 1 of the reaction in ethanol as the solvent at 293.15 k.

Solvent	Ethanol (24.7 ^a^)
k_1_ (M^−1^·S^−1^)	1.02 × 10^2^
Completion time (s)	20

^a^ Dielectric constant (D).

**Table 2:** The values of k_obs_ (min^−1^) for the reaction between TPP (5.00 × 10^−3^ M), DMAD (5.00 × 10^−3^ M) and MA (5.00 × 10^−3^ M) in different solvent media and temperatures.

**Temperature**	**Solvent: Ethanol (24.7) ^a^**		**Temperature**	**Temperature**
T	T = 288.15 K	T = 293.15 K	T = 298.15 K	T = 303.15 K
k_obs_	0.750 (0.002) ^b^	0.870 (0.002)	0.990 (0.002)	1.14 (0.002)
	**Solvent: 1,2-Dicholoroethan (10.37) ^a^**			
T		T = 293.15 K		
k_obs_		3.65 (0.004)		

^a^ Dielectric constant (D). ^b^ Standard deviation (SD).

**Table 3:** Activation parameters for the reaction between compounds TPP, DMAD and MA measured in the solvent ethanol.

Solvent	ΔH^‡^ (kJ·mol^−1^)	ΔS^‡^ (J·mol^−1^)	TΔS^‡^ (kJ·mol^−1^)	ΔG^‡^ (kJ·mol^−1^)	Ln A	Ea (kJ/mol)
Ethanol (24.7) ^a^	17.72 ± 0.07 ^b1^	−185.49 ± 0.03 ^b1^	−54.38 ^b1^	71.17 ± 0.02 ^b1^	8.14 ^c^	20.16 ^c^ 20.17 ^d^
	17.72 ± 0.05 ^b2^	−185.49± 0.02 ^b2^	−54.38 ^b2^	72.09 ± 0.01 ^b2^		

^a^ Dielectric constant (D). ^b1^ According to Equation (9). ^b2^ According to Equation (8). ^c^ According to this equation: Ea = ΔH^‡^ + RT. ^d^ In accord with the Arrhenius equation.
